# KANK1 inhibits cell growth by inducing apoptosis though regulating CXXC5 in human malignant peripheral nerve sheath tumors

**DOI:** 10.1038/srep40325

**Published:** 2017-01-09

**Authors:** Zhibin Cui, Yingjia Shen, Kenny H. Chen, Suresh K. Mittal, Jer-Yen Yang, GuangJun Zhang

**Affiliations:** 1 Department of Comparative Pathobiology, Purdue University, West Lafayette, Indiana, United States; 2 A316 Environment and Ecology Building, Xiamen, Fujian 361102, China; 3 Purdue University Center for Cancer Research, West Lafayette, Indiana, United States; 4 Purdue Institute for Inflammation, Immunology and Infectious Diseases (PI4D), West Lafayette, Indiana, United States; 5 Department of Basic Medical Sciences, Purdue University, 625 Harrison Street, West Lafayette, Indiana, 47906, USA; 6 Integrative Neuroscience Center; Purdue University, 625 Harrison Street, West Lafayette, Indiana, 47906, USA

## Abstract

Malignant peripheral nerve sheath tumors (MPNSTs) are a type of rare sarcomas with a poor prognosis due to its highly invasive nature and limited treatment options. Currently there is no targeted-cancer therapy for this type of malignancy. Thus, it is important to identify more cancer driver genes that may serve as targets of cancer therapy. Through comparative oncogenomics, we have found that *KANK1* was a candidate tumor suppressor gene (TSG) for human MPNSTs. Although *KANK1* is known as a cytoskeleton regulator, its tumorigenic function in MPNSTs remains largely unknown. In this study, we report that restoration of *KANK1* in human MPNST cells inhibits cell growth both in human cell culture and xenograft mice by increasing apoptosis. Consistently, knockdown of *KANK1* in neurofibroma cells promoted cell growth. Using RNA-seq analysis, we identified *CXXC5* and other apoptosis-related genes, and demonstrated that *CXXC5* is regulated by KANK1. Knockdown of *CXXC5* was found to diminish KANK1-induced apoptosis in MPNST cells. Thus, KANK1 inhibits MPNST cell growth though CXXC5 mediated apoptosis. Our results suggest that *KANK1* may function as a tumor suppressor in human MPNSTs, and thus it may be useful for targeted therapy.

MPNST is a sarcoma derived from Schwann cells, and accounts for 3–10% of all soft-tissue sarcomas^1^. Surgical resection is the mainstay of MPNST therapy, but its prognosis remains poor due to invasive growth, metastasis, and insensitivity to radiotherapy and chemotherapy^2^,^3^. In human populations with MPNST, about half of the patients have familial *NF1* gene mutations, while the other half appear to have sporadic gene mutations^4^,^5^. Both of the NF1-associated and sporadic MPNST patients have been found to possess very similar aneuploid chromosomes and DNA copy number alterations (CNAs)^2^,^3^,^6^,^7^. Certain aneuploid chromosomes are known to be highly correlated with tumorigenesis and patient survival^3^. Chromosome 9p is one of the most frequently underrepresented chromosome arms in MPNSTs, along with many other solid tumors^8^,^9^,^10^, suggesting it carries important TSGs.

Identifying TSGs on lost aneuploid chromosomes is difficult, as there are usually many genes associated with them. Cross-species comparative oncogenomics has recently emerged as a new approach to identify cancer driver genes (TSGs and oncogenes)^11^,^12^,^13^. In zebrafish, MPNSTs can be modeled by either *ribosomal protein (rp)* genes or *tp53* mutations^14^,^15^. We have previously found that MPNSTs from both types of mutations share almost identical CNAs, and that zebrafish MPNSTs are highly aneuploid containing a similar number of CNAs to those in human cancers^16^,^17^. Using zebrafish-human comparative oncogenomic analysis on CNAs of both zebrafish and human MPNSTs, we identified *KANK1*, as a candidate TSG on chromosome 9p.

Except known as a gene for cerebral palsy spastic quadriplegia type 2^18^, *KANK1* has been reported as a candidate TSG in renal cell carcinoma patients, as it was found that KANK1 re-expression was able to inhibit HEK293 cell growth by reducing proliferation^19^. *KANK1* mutations were also associated with myeloproliferative neoplasm, and a fusion protein of KANK1 with PDGFRB was found as an oncogene due to a t(5:9) translocation^20^. Although *KANK1* alterations are frequently found in many solid tumors including MPNST, its detailed cellular and molecular mechanisms on tumorigenesis remain largely unknown, except, that it is able to regulate actin polymerization and cell migration through RAC1 and RHOA signaling^21^,^22^.

Apoptosis is a common pathway of programmed cell death, and its dysregulation is seen in a variety of human pathologies, including cancers. In this paper, we report that KANK1 positively regulates apoptosis and inhibits cell growth in human MPNST cells. Using RNA-seq, we identified a new KANK1 downstream gene, *CXXC-type zinc finger protein 5 (CXXC5*), which was recently reported as a mediator for TNF-α apoptosis pathway, and is involved in acute myeloid leukemia^23^,^24^. Knockdown of *CXXC5* diminished KANK1-induced apoptosis, suggesting CXXC5 is one of the key effectors of KANK1. Overall, our results suggest that *KANK1* might function as a new TSG in human MPNSTs.

## Results

### DNA copy numbers of KANK1 are frequently lost in both human and zebrafish MPNSTs

The *KANK1* gene is a potential tumor suppressor gene located at 9p42.3, a chromosomal segment, which is generally under-represented in more than half of human MPNSTs^17^ (Supplementary Fig. S1a). In zebrafish, there are two *kank1* genes (*kank1a* and *kank1b*) due to the teleost whole genome duplication^25^. Both *kank1* genes are located on zebrafish chromosome 5, which is also lost in most zebrafish MPNSTs (Supplementary Fig. S1b). TSGs may lose their functions through multiple mechanisms such as nucleotide mutation and gene expression downregulation. To further explore the mutation nature and scope of *KANK1* locus deletions, we analyzed human cancer genomic data using cBioportal^26^. Indeed, *KANK1* is deleted in ~20% MPNSTs in the Sloan Kettering data set (3 deep-deletion and 5 shallow-deletion out of 15 samples). Moreover, KANK1 deletions and mutations (missense, nonsense, and frameshift mutations) are also frequent in prostate, lymphoid, pancreatic, uterine, and stomach cancers (Supplementary Fig. S1c). These results are consistent with reported deletions of *KANK1* in a variety of human cancers including MPNSTs^2^,^17^,^27^.

### KANK1 negatively regulates cancer cell growth in human MPNST cells

**Figure 1:**
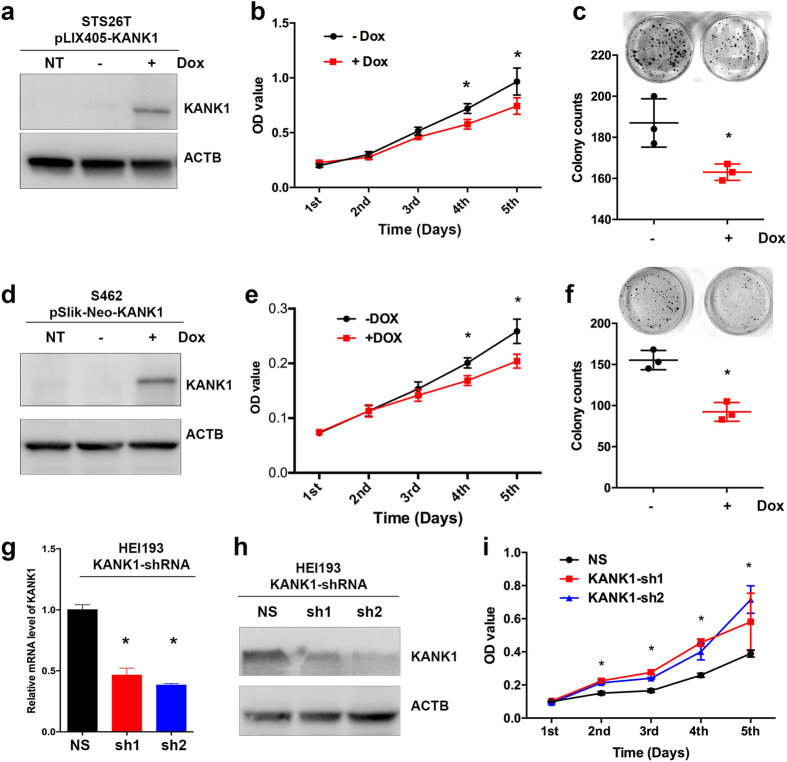
Restoration of *KANK1* in human MPNST cells suppresses cell growth in cell culture. KANK1 gene expression was restored in lentiviral *KANK1* stable STS26T (**a–c**) and S462 (**d–f**) cells using doxycycline. (**a**,**d**) Restoration of KANK1 measured by Western blots in both lines (STS26T & S462). The lentiviral constructs pLIX405-*KANK1* and pSLIK-neo-*KANK1* are labeled above the Western blot gels. NT, non-transfected. ACTB, beta-actin control. (**b**,**e**) MTT assays revealed that cell growth was inhibited by *KANK1* restoration in both cells. Growth curves were created using the optical density values collected over five days. (**c**,**f**) Plate colony formation assay on the STS26T and S462 cells with or without *KANK1* restoration. Crystal violet stained cell colonies (diameter ≥0.2 mm) were counted. (**g**,**h**) HEI-193 cells with *KANK1* knocked-down using shRNAs were confirmed by QRT-PCR (**g**) and Western blot (**h**). NS, non-silencing; sh1 and sh2, *KANK*1-shRNAs. (**i**). MTT assays showed that *KANK1* knockdown is able to promote cell growth. Asterisk (*) shows statistical significance, p < 0.05.

DNA copy number loss usually leads to low gene expression levels^28^, therefore we reasoned that restoration of KANK1 in human MPNST cells will reduce cell growth rate if *KANK1* functions as a TSG. As *NF1* and *TP53* are commonly known genes that are involved in human MPNSTs, we chose both STS26T (*TP53* mutant) and S462 (*NF1* mutant) human MPNST cell lines^29^ for our studies. To avoid artificial high expression of KANK1, we choose tetracycline-inducible lentiviral system that allows us to tightly control the level of gene expression. Both C-terminal GFP tagged (pLIX405-KANK1) and untagged full-length KANK1 (pSlik-neo-KANK1) constructs were created for the generation of stable cell lines (Fig. 1a,d). As expected, KANK1 expression was positively correlated to doxycycline dosages, and the level was reversed to undetectable levels upon withdrawing doxycycline treatment (Supplementary Fig. S2a–c). To assess the impact of KANK1 restoration, we first measured overall cell growth using MTT assay. Upregulation of KANK1 expression resulted in a decrease of cell growth rates in both MPNST cell lines (Fig. 1b,e). The cell growth inhibition corresponds to the dosage increase of the doxycycline (Supplementary Fig. S2d). As doxycycline may have side effects on overall cell growth, we performed MTT assays on non-transfected cells with or without doxycycline treatment. No difference was observed between the two groups, even with a higher concentration of 5 μg/ml (Supplementary Fig. S3). Consistent with our MTT assay, we found that KANK1 upregulation led to a reduction of colony formation numbers (Fig. 1c,f), suggesting that KANK1 is able to reduce independent growth capacity, a key characteristic of human cancers. As cancer cells usually survive in a stressful environment *in vivo*, we reasoned that restoring KANK1 in such stressful condition might give more insight to its function. Indeed, we observed a more remarkable difference in the numbers of colony formation between the control and KANK1-expressing groups in low serum condition (0.5% FBS) (Supplementary Fig. S2e). To further investigate the tumorigenic roles of KANK1, we knocked down *KANK1* expression in an immortalized benign schwannoma cell line, HEI-193^30^, using shRNAs against *KANK1*. Consistently, there was an increase of cell growth rate in *KANK1*-knocked-down cells compared to the non-silencing control (Fig. 1g–i).

### KANK1 negatively regulates cancer cell growth in mouse

**Figure 2:**
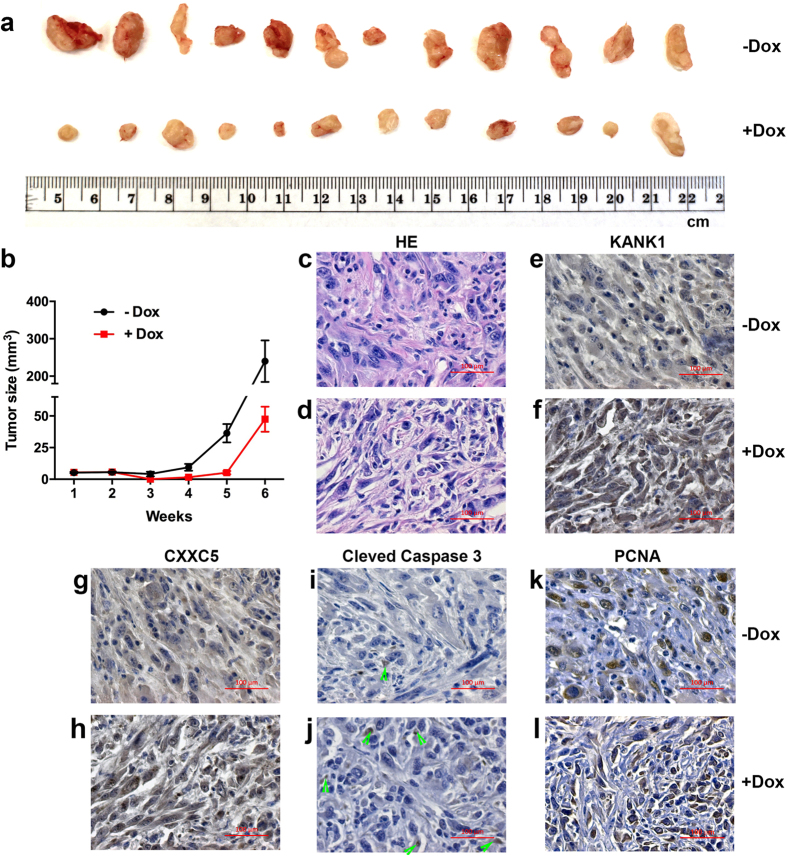
*KANK1* inhibits MPNST growth in xenografted mice. (**a**) Xenografted human MPNST STS26T cells in NOD/SCID mice with (+Dox) and without (-Dox) doxycycline feeding (200  μg/ml). (**b**) Changes in tumor volume over time. p < 0.001 (two-way ANOVA). (**c**,**d**) MPNST histology by hematoxylin and eosin (HE) staining. (**e**,**f**) KANK1 protein in STS26T cells measured by immunohistochemistry. (**g**,**h**) CXXC5 protein in STS26T cells measured by immunohistochemistry. (**i**,**j**) Cleaved caspase 3 measured by immunohistochemistry. Green arrow heads indicate the positively stained cells. (**k**,**l**) PCNA, a proliferation maker, measured by immunohistochemistry. (**e**–**l)** Slides were counter stained with hematoxylin. A scale bar is on the right-bottom corner for each panel (**c**–**l**).

To further demonstrate cell growth inhibitory roles of the *KANK1* gene, we turned to a xenograft mouse model. The tetracycline-inducible KANK1 stable cells were subcutaneously inoculated into NOD/SCID mice. One week after inoculation, KANK1 expression was induced by doxycycline. The size of the inoculation bumps were tracked twice a week by measuring the diameters. We did not detected any differences between doxycycline treated and untreated mice during the first three weeks. However, the size differences of tumors in the doxycycline treated and untreated groups were noticeable from the fourth week. The tumor size differences became more evident in the fifth and sixth weeks (Fig. 2a,b). By the end of week six, tumor volumes in the doxycycline-induced KANK1 expressing mouse group were significantly smaller (Fig. 2a,b). The animals were euthanized and tumor tissues were collected and processed for histopathology and immunohistochemistry. HE staining of the tumor sections confirmed the characteristics of MPNST. As expected (Fig. 2c,d), the KANK1 protein expression level was higher in the tumors of doxycycline-treated mice (Fig. 2e,f). These results demonstrated that KANK1 inhibits human MPNST cell growth in the mouse xenograft model.

### *KANK1* gene restoration inhibits cell growth by inducing apoptosis

**Figure 3:**
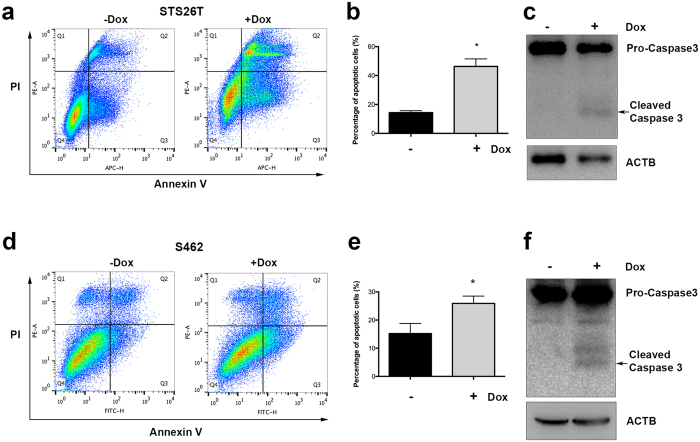
KANK1 inhibits cell growth through apoptosis. (**a**) Representative of three experiment replicates with STS26T. KANK1 expression cells were prepared with (+Dox) and without (−Dox) doxycycline at 1 μg/ml in culture medium. Apoptosis was analyzed with FlowJo after Fluor 647 conjugated Annexin V and PI double-staining flow cytometry. Quadrant I-IV represents dead cell population, early apoptotic cell population, late apoptotic cell population, and live cell population, respectively. (**b**) Statistical analysis showed significant difference between KANK1 expressing and control groups in STS26T. Asterisk (*) shows p = 0.0161. (**c**) KANK1 restoration induced apoptosis measured by cleaved caspase 3 expression. Both control and doxycycline-treated cells were cultured at medium with 0.5% FBS. ACTB, beta-actin control. (**d**) Representative of three experimental replicates with S462 cell line. Flow cytometry was formed using Fluor 488 conjugated Annexin V and PI double staining. (**e**) Statistical analysis showed significant difference between KANK1 expressing and control groups. Asterisk (*) shows statistical significance, p = 0.0087. (**f**) KANK1 restoration induced apoptosis measured by cleaved caspase 3 expression.

Cell growth inhibition could be caused by either slow cell proliferation or increased cell death. In HEK293 cells, it was found that KANK1 was able to induce G_0_/G_1_ arrest upon KANK1 expression^19^. To elucidate the cellular mechanism of KANK1 inhibition on MPNST cells, we first analyzed cell cycle alterations using BrdU pulse S-phase labeling. Contrast to previous reports in HEK293 cells^19^, we did not find statistically significant differences between doxycycline-induced and untreated cells for any cell cycle phases in both 10% and 0.5% FBS conditions from day 2 to day 4 (Supplementary Fig. S4). Next, we went on to analyze apoptosis. As expected, we found increased apoptotic cell population in the doxycycline-induced cells using flow cytometry analysis with Annexin V staining in 0.5% FBS culture condition (Fig. 3a,b,d,e). To verify the increase in apoptosis, we performed Western blot analysis using anti-caspase 3 antibody, which was able to detect both procaspase 3 and cleaved caspase 3. Consistently, cleaved caspase 3 levels were found higher in doxycycline-treated MPNST cells compared to untreated controls (Fig. 3c,f). Similar with the increased apoptosis in cell culture, the cleaved caspase 3 was detected in the xenograft KANK1 expressing STS26T cells in mice (Fig. 2i,j, and Supplementary Fig. S5), while no statistical significant change was found with the cell proliferation marker, PCNA (Fig. 2k,l). To further verify apoptosis, we examined cell nucleus morphology using Hoechst 33342 staining. More condensed nuclei were found in the doxycycline treated cells (Supplementary Fig. S6). All these results demonstrate that KANK1 inhibits cell growth through apoptosis.

### Identification of *CXXC5* as one of the KANK1 downstream genes

**Figure 4:**
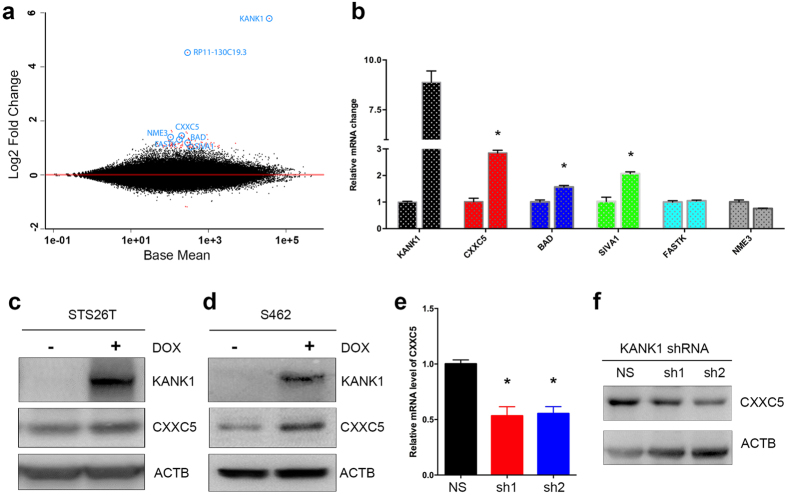
Identification of *CXXC5* as a downstream gene of KANK1. (**a**) Differential gene expression by RNA-seq before and after *KANK1* restoration. Each dot represents the mean expression level plotted against the fold change for a given transcript. Black points are transcripts that are not statistically significant, red points are significant at FDR 10% (adjusted p-value < 0.1), and fold change is greater than 2. *KANK1* and the five apoptosis-related genes are labeled with blue circles. The RP11-130C19.3 is a putative transcripts within *KANK1* gene locus according to the current Ensembl assembly. (**b**) Quantitative RT-PCR validations for *KANK1* and the five apoptosis related genes using the same RNA samples for RNA-seq. Asterisk (*) indicates statistical significance: p = 0.0004 (*CXXC5*), 0.0039 (*BAD*), and 0.0039 (*SIVA1*). (**c**,**d**) CXXC5 protein levels increase when KANK1 is restored in both STS26T and S462 stable cells using doxycycline. ACTB, beta-actin control. (**e**,**f**). *CXXC5* is down-regulated in *KANK1* knock-down HEI-193, by both QRT-PCR (**e**) and Western blot (**f**). NS, stable cell line made using non-silencing shRNA. Asterisk (*) shows statistical significance: p = 0.0065 (NS vs. sh1) and p = 0.0035 (NS vs. sh2).

Apoptosis is one of the common pathways for programed cell death, and it involves many genes^31^. To better understand gene expression changes in KANK1-expressed cells, and how KANK1 interacts with apoptosis machinery, we employed RNA-seq analysis. To detect early gene expression alterations, and to avoid late changes resulting from apoptosis, we chose to examine our cells at 48 hours after doxycycline treatment, since the observed cell growth inhibition effect was usually overt around the fourth day (Fig. 1b,e). Three replicates of doxycycline induced and un-induced cells were sequenced. About 30 million 100 bps paired-end reads per sample were generated and mapped to the reference human genome GRCh38.p5. After quantifying and comparing gene expression levels, we identified 3,578 genes that were differentially expressed in doxycycline-induced KANK1 expressing cells versus control cells. Among these genes, 2,144 genes were upregulated and 1,434 genes were downregulated (Supplementary File S1). For most of the differentially expressed genes, the alteration levels were mild (less than 2 fold). We only identified 88 annotated genes whose expression levels were changed more than two-fold. Eighty five of them were upregulated, and two microRNAs (mir-423 and mir-3184) were downregulated (Fig. 4a and Supplementary File S1). To identify key genes related to apoptosis, we performed literature searches for genes that have been reported to regulate apoptosis. We identified 5 genes: *CXXC5, NME3, BAD, SIVA1* and *FASTK* (Fig. 4a). Using quantitative RT-PCR (QRT-PCR), we were able to confirm changes in expression levels of three out of these five genes in an additional set of KANK1-inducible cells (Fig. 4b). Among these three genes, *CXXC5* mRNA levels were most significantly upregulated upon *KANK1* induction (Fig. 4a,b). Moreover, an increase of CXXC5 at the protein level was detected in both KANK1-inducible STS26T and S462 cells (Fig. 4c,d). To further confirm that CXXC5 is regulated by KANK1, we first examined CXXC5 protein when KANK1 is overexpressed in HEK293T cells, and found CXXC5 protein was increased when KANK1 was expressed (Supplementary Fig. S7). Consistent with our finding that KANK1 can induce CXXC5 expression, we found that CXXC5 protein level was lower in KANK1 stable knockdown HEI-193 cells, compared to the non-silencing control cells (Fig. 4e,f). Lastly, we also detected upregulated CXXC5 protein in the doxycycline-induced mouse xenografted tumors (Fig. 2g,h), suggesting that CXXC5 acts in a downstream manner to KANK1 *in vivo*.

### CXXC5 is one of the mediators of KANK1 induced apoptosis

**Figure 5:**
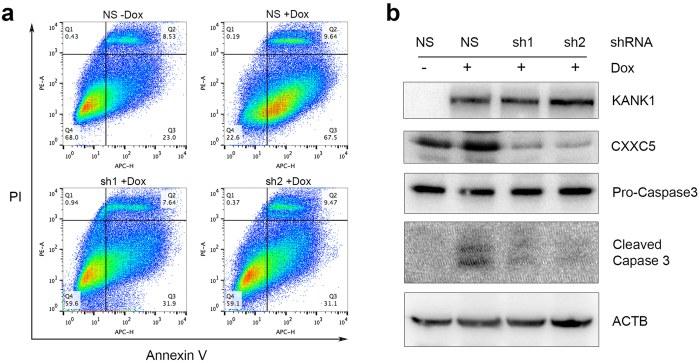
CXXC5 knockdown diminishes KANK1 induced apoptosis. (**a**) Apoptosis measured by flow cytometry using Annexin V and PI in *CXXC5* shRNA stable STS26T cells, and non-silencing control shRNA cells. Quadrant I-IV represents dead cell population, early apoptotic cell population, late apoptotic cell population, and live cell population, respectively. Later apoptotic cell percentage was reduced from 67.5% to ~31% in both *CXXC5* shRNAs infected cells. (**b**) *CXXC5* knockdown rescued KANK1-induced apoptosis, which is evident by reduced expression of cleaved caspase 3. NS, non-silencing shRNA. sh1 and sh2, *CXXC5* shRNAs. Dox, doxycycline (1 μg/ml).

CXXC5 was recently reported as a player in regulating apoptosis^23^. Based on our results of RNA-seq and KANK1-mediated upregulation of CXXC5, we reasoned that CXXC5 might be a key mediator for the KANK1-induced apoptosis. To confirm this, *CXXC5* knockdown stable cell lines were generated on top of KANK1-inducible stable cells using anti-*CXXC5* shRNAs reported previously^24^. The CXXC5 knockdown efficiency was confirmed by Western blot (Fig. 5). To elucidate whether CXXC5 is required for KANK1-induced apoptosis, we examined apoptosis in the CXXC5 knockdown cell lines with or without doxycycline treatment. With flow cytometry using Annexin V staining, we found apoptotic cells were reduced in the *CXXC5* knockdown cells compared to the non-silencing control under the condition of KANK1 restoration (Fig. 5a). We were also able to confirm that CXXC5 silenced cells had lower levels of cleaved caspase3 (Fig. 5b). Furthermore, we performed immunohistochemistry on the xenografted MPNST tumors, and found a higher level of CXXC5 protein expression and more apoptotic cells in KANK1-expression tumors from doxycycline treated mice (Fig. 2g–j). Together, our results demonstrated that CXXC5 is one of the downstream mediators in KANK1-induced apoptosis.

## Discussion

Although MPNST is a rare disease in humans, it represents 3–10% of all soft-tissue sarcomas^1^. The prognosis of this malignancy is poor due to its invasive nature and limited treatment options. Currently, there are no targeted therapies available mainly because of the lack of suitable targets, which are usually cancer driver genes. In this study, we showed that KANK1 inhibits cell growth by inducing apoptosis upon restoring *KANK1* expression in human MPNST cells. Using RNA-seq, an unbiased analysis, we identified *CXXC5* as a KANK1 downstream gene and demonstrated that *CXXC5* is a mediator for KANK1-induced apoptosis. Our results not only suggest that *KANK1* may function as a TSG, but also reveal a new molecular mechanism on how *KANK1* regulates apoptosis.

### *Roles of KANK1* as a growth suppressor through apoptosis

*KANK1* has been reported as a growth suppressor in human HEK293 cells, and ectopic expression caused G_1_/G_0_ arrest after transient transfection^19^. Similarly, a recent study with glioma cell lines confirmed that *KANK1* transient re-expression was able to block cells in the G_1_/G_0_ phase^32^. Although cell growth suppression is consistent with previous reports, G_1_/G_0_ cell cycle arrest was not seen in our doxycycline-inducible stable MPNST cells. We did not detect any statistically significant alterations in different phases of the cell cycle, even with BrdU labeling (Supplementary Fig. S4). One possible explanation for the differences is due to different types of cells, but more likely this discrepancy might result from the level and duration of *KANK1* expression. Instead of transient transfections, we employed a doxycycline-inducible system. Moreover, we chose 1 μg/ml, a relatively low dose of doxycycline to induce KANK1 expression (Fig. S2). Thus, the artificial effects of high level of gene expression is controlled. As overall cell growth inhibition can be achieved through either a slower proliferation rate (G2/M reduction), or an increased rate of cell death, we hypothesized that cell death may explain *KANK1*’s growth suppression. Indeed, we were able to detect increased apoptosis in the KANK1*-*induced MPNST cells. In addition, higher levels of cleaved caspase 3 was detected in our mouse xenograft tumors, suggesting that *KANK1* restoration is able to induce apoptosis *in vivo*. Since both STS26T and S462 showed similar responses to *KANK1* restoration, and the two cell lines possess *NF1* or *TP53* mutations, respectively^29^, *KANK1* most likely induced apoptosis in a TP53-independent/NF1-independent manner. This is particularly important considering the high frequency of *TP53* and *NF1* mutations in human cancers. In a recent study with nasopharyngeal cancer cell lines, epigenetically silenced *KANK1* was reactivated by the demethylating agent Decitabine (5-aza-2′-deoxycytidine)^33^. The re-expressed *KANK1* by 5-aza-CdR treatment can reduce cell proliferation and increase apoptosis^33^, suggesting KANK1 might be an option for MPNST therapeutics.

### *CXXC5* mediates *KANK1* cell growth inhibition

Currently, on the molecular level it is known that KANK1 interacts with KIF21A, BAIAP2 (IRSP53), 14-3-3 s, BIG1, beta-catenin, and Liprin β1, and regulates actin polymerization and cell migration^21^,^34^,^35^,^36^,^37^. However, none of these signaling pathways except beta-catenin directly explains apoptosis induced by *KANK1*. To elucidate the molecular pathways in which KANK1 induces apoptosis, we performed RNA-seq, and identified *CXXC5* as one of the KANK1-induced apoptosis genes. CXXC5 is a member of the protein family that contains a ZF-CxxC domain, which is characterized by two conserved cysteine-rich clusters that bind two Zn^2+^ ions^38^. *CXXC5*, also known as *RINF* (retinoid-inducible nuclear factor), was first identified as an important gene for human myelopoiesis, and is frequently lost in myeloid leukemia^24^. *CXXC5* can be induced by BMP signaling and interacts with Dishevelled (Dvl), a Wnt-signaling intermediator^39^. It has been reported to be involved in an apoptosis pathway^23^, and the higher CXXC5 expression level was associated with increased apoptosis in primary leukemia^40^. This is consistent to our observation that KANK1-induced apoptosis is partially mediated by CXXC5. KANK1 is known to be a shuttle protein for beta-catenin’s nucleo-cytoplasmic translocation^36^, additionally beta-catenin is known to be able to bind to the *CXXC5*′s promoter^41^, therefore, it is likely that KANK1 and beta-catenin bind directly to the promoter of *CXXC5*, and regulate its expression at the transcriptional level. Furthermore, since knockdown of *CXXC5* alone did not completely abrogate KANK1 induced apoptosis, it will be interesting to identify other mediator genes downstream of *KANK1*, such as *SIVA1* and *BAD*, in the future.

Overall, our experimental results provide evidence for *KANK1* as a tumor suppressor in human MPNST cells, and elucidate a new mechanism of *KANK1* cell growth suppression. Additional animal models of KANK1 are needed for further evaluating *in vivo* tumor suppressor functions of KANK1. We have already created a few loss-of-function zebrafish *kank1a* and *kank1b* mutants using CRISPR, and are currently investigating *KANK1’*s tumor suppressor capacity.

## Materials and Methods

All methods were carried out in accordance with relevant guidelines and regulations. All experimental protocols using cell lines, plasmid constructs, and animal manipulation protocols were approved by Purdue University institutional review board (IRB protocol # 12-029). NOD/SCID/IL2RG^Null^ mice used in this study were purchase from Jackson Laboratory and maintained at the Purdue animal housing facility, which is an AAALAC-approved animal facility. All animal related experiments were carried out according to the protocols approved by the Purdue Animal Care and Use Committee (PACUC) (Protocol # 1301000800).

### Cell lines and cell culture

The human malignant peripheral nerve sheath tumor cell lines, STS26T and S462, were kindly provided by Dr. George De Vries, and Dr. Karen Cichowski, respectively. The immortalized neurofibroma cell line HEI-193^30^ was purchased from ATCC, American Type Culture Collection. All three cell lines were authenticated by ATCC using short tandem repeat profiling. Cells were grown in DMEM with 10% heat inactivated fetal bovine serum (FBS), penicillin (100 IU/ml), and streptomycin (100 μg/ml). For the low serum culture condition, all medium components remain the same except 0.5% FBS was used instead of 10% FBS. All cell cultures were carried out at 37 °C in a humidified 5% CO_2_ atmosphere.

### Plasmid constructs

The *KANK1* cDNA without a stop codon in pENTR223 was purchased from DNASU (HsCD00516496). A full-length sequence of *KANK1* with a stop codon was amplified by PCR using the purchased clone as a DNA template, and sub-cloned into pEN_TTmcs (Addgene #25755), pLX301, and pSLIK-Neo, which were purchased from Addgene (#25895 and #25735). pLIX405 was modified from pLIX403 (Addgene #41395) by replacing the V5 tag with EGFP. The pSlick-Neo-*KANK1*, pLIX405-*KANK1*, and pLX301-KANK1 were created using Gateway LR Clonase II. *KANK1* shRNA V2LHS-50967 (KANK1-sh-1), *KANK1* shRNA V2LHS-50970 (KANK1-sh-2), and a non-silencing (NS) control GIPZ plasmid (RHS4346) were purchased from GE Dharmacon. *CXXC5* shRNA TRCN0000144558 (*CXXC5*-sh-1), TRCN0000142729 (*CXXC5*-sh-2), and a non-silencing control (NS) pLKO.1-vector were purchased from Sigma-Aldrich.

### Stable cell line generation

Lentiviral particles were prepared in HEK293T cells by co-transfecting the *KANK1* cDNA or shRNA plasmid with pCMV-VSV-G and pCMV-dR8.2. Culture medium containing lentiviral particles was harvested 48–72 hours post transfection to subsequently infect MPNST and neurofibroma cells. At 48 hours after infection, cells were subject to puromycin (2 μg/ml) or G418 (200 μg/ml) selection for about 2 weeks to establish stable cells. Western blot and QRT-PCR were performed to test gene expression or knocked-down cells.

### MTT cell growth assay

Tumor cells were seeded into 96-well plates with 1000 cells in each well, and cultured in a 37 °C incubator overnight. In the experimental wells, 1 μg/ml doxycycline was added to induce KANK1 expression. On the second day, cells were washed twice with PBS, and incubated with MTT (0.5 mg/ml) for 4 hours. Then, crystalized purple-colored formazan in cells was dissolved by adding 100 μl DMSO per well. Optical density at 595 nm was recorded using a plate spectrophotometer (Bio-TEK). For each cell line or experimental condition, at least 3 replicates were performed at each time point.

### Plate colony formation assay

For each treatment condition, 1,000 cells were seeded into a 6 cm diameter cell culture plate, and incubated for 24 hours at 37 °C. Fresh medium with doxycycline (1 μg/ml) was changed every 3 days to maintain constant KANK1 expression. Three weeks later, cells were fixed and stained with 0.25% crystal violet solution. After washing the plates with water, the cell colonies were imaged and counted using GelDoc-IT2 imaging system (UVP). Cell colonies with a diameter of more than 0.2 mm were counted as positive dots.

### Western blotting and antibodies

All treated cells were harvested and lysed using RIPA buffer with 0.5 mM PMSF. Cell debris was removed by centrifuge before quantification using Bradford Protein Assay Kit (IBI Scientific). About 20–50 μg of protein was loaded per well on 8–15% SDS-PAGE gel, depending on the expected protein sizes. Proteins in gels were then transferred to polyvinylidene fluoride membranes, which were subject to immunoblotting. Signals were detected using ECL Western Blotting Substrate Kit (Pierce). Images were collected using G:Box XR5 imaging system (Syngene). Primary antibodies: KANK1 (Santa Cruz, sc-135113), CXXC5 (Proteintech, 16513-1-ap), Caspase 3 (Santa Cruz, sc-7148), and ACTB (Santa Cruz, sc-47778). Secondary antibodies: anti-mouse (Proteintech sa00001-1) and anti-rabbit (Proteintech sa00001-2).

### Flow cytometric analyses

Cells were prepared and cultured at least two days before each experiment. To analyze apoptosis, the Annexin V dead cell apoptosis kit (ThermoFisher) was used following the manufacturer’s instruction. Flow cytometric analyses was performed using FACSCanto II. For cell cycle analysis, BrdU was employed to label the cells in S phase. Briefly, cells were incubated with 10 μg/ml BrdU for 30 minutes, and then fixed in 75% ethanol. The cells were treated with 3 N HCl with 0.5% Triton X-100 for 20 minutes to denature the gDNA and permeabilize the cells. Then, the cells were neutralized with 0.1 M Na_2_B_4_O_7_ pH8.5. Finally, the cells were stained with anti-BrdU, and secondary antibody conjugated with Alexa Fluor^®^ 647. The cells were run through a flow cytometer, and data were analyzed using FlowJo.

### Immunohistochemistry (IHC) staining and antibodies

IHCs were performed using Vectastain Elite ABC kit (Vector Laboratories) according to the manufacturer’s instruction. Xenograft tumors from mice were imbedded in paraffin, and tissue sections on slides were conducted by the Purdue Histology and Phenotyping Laboratory. All the stained sections were counterstained with Mayer’s hematoxylin. Protein expression was imaged with Zeiss Axio Imager 2 microscopy. Antibodies used for the detection of proteins were the same as the ones used for Western blotting.

### Cell nucleus morphology

About 1 × 10^5^ cells were plated and cultured with or without doxycycline in a 12-well plate. After 48 hours, cell cultural medium was removed, and cells were washed in PBS three times. Cells in PBS with Hoechst33342 (Sigma, B2261) at the concentration of 5 ug/ml was added, and were incubated for 10 minutes at room temperature. Then, staining solution was removed, and cells were washed with PBS three times before imaging. Cells were visualized and imaged using a fluorescence microscope (DMi8, Leica, USA) with a DAPI filter (450 nm). Cells with condensed nuclei were counted as apoptotic cells. Three microscopic views were analyzed for each condition. Apoptotic rates in both doxycycline treated and untreated groups were calculated with student t-test.

### Mouse xenograft experiment

About 1 × 10^7^ STS26T cells (STS26T-pLIX405-KANK1) were mixed with matrigel, and injected subcutaneously in four sites on each mouse (shoulder and upper hind legs of both sides). In total, six mice were injected. Control mice were given fresh water daily, while experimental mice were given water containing 200 μg/ml doxycycline beginning at the second week after inoculation. Tumor growth was monitored and/or measured every week until the end of the experiment. Mice were euthanized at end of the sixth week. Tumors were then dissected for volume analysis. For the tumor from each injection site, the length and width of each tumor was measured. The tumor volume was calculated using the formula, V = 1/2 × A × B^2^ (V, tumor volume; A, length in millimeter; B, width in millimeter). Tumor tissues were then fixed with 4% PFA overnight for pathological analysis.

### RNA-seq library preparation and bioinformatics

Three replicates of cells from stable cell line STS26-pLIX405-KANK1 were prepared with or without 1 μg/ml doxycycline treatment for 48 hours. Total RNA was extracted from these cells using AllPrep DNA/RNA/Protein Mini Kit (Qiagen, 80004) following the manufacturer’s instruction. For each sample, 2 μg total RNA was used for RNA-seq library preparation.

Sequencing libraries were constructed from the total RNA using the Illumina TruSeq Stranded mRNA Library Prep Kit mostly as directed by the kit’s direction, but with some modifications. Fragmentation time in the elute-fragment-prime step was reduced from 8 to 4 minutes, resulting in cDNA ranging in length from 100 to 1000 bps. Subsequent AMPure purification was performed at a sample-to-AMPure ratio of 1.8:1 (v/v). The resulting libraries were composed of amplicons largely ranging in length from 200 to 1000 bps. Furthermore, eight cycles of PCR amplification were undertaken rather than the specified 15 cycles. Individual libraries were pooled based on titer using KAPA Library Quantification on an Applied Biosystems Step-One qPCR machine. A single pool of all 6 libraries was clustered on one lane of a HiSeq 2500 High Output 2 × 100 base read chemistry run.

For RNA-seq analysis, we first pre-processed RNA-seq reads using a custom Perl script to remove unreliable reads, and to trim sequences with low quality sections of each read based on following the filtration algorithm as described previously^42^. Any reads with bases uncalled and phred quality score of 2 were rejected. Reads were then trimmed where the quality score value was under 10, or the average score of three continuous bases was under 20. We then mapped the processed reads against the reference human genome (GRCh38) using Tophat2^43^. To quantify number of read counts per transcript, we employed HTSeq^44^ with genome annotation downloaded from Ensembl (release 81), and preformed differential expression analyses using DESeq2 package^45^. Genes with FDR (false discovery rate) less than 10% (adjusted p-value < 0.1), and more than a two-fold change was considered as significant. The RNA-seq original data reported in this paper have been deposited in the NIH Gene Expression Omnibus (GEO) database (accession #. GSE85271).

### Quantitative RT-PCR

Total RNAs were isolated from cells using TRIzol reagent according to the manufacturer’s instruction. For reverse transcription, 2 μg total RNA was used as a template, and cDNAs was synthesized using the Transcriptor First Strand cDNA Synthesis Kit (Roche). QRT-PCRs were conducted using SYBR Green I Master Mix (Roche), following the manufacture’s instruction on Light Cycler 480. Primers (Supplementary Table 1) for each gene were designed to cover all the transcripts, which are located on different exons to avoid potential genomic DNA contamination. PCRs were performed at the following condition: 95 °C, 10 seconds; 60 °C, 15 seconds; and 72 °C, 20 seconds for 40 cycles. Results were analyzed using ΔCt method to calculate relative gene mRNA level^46^.

### Statistical analysis

All statistical analyses were performed using GraphPad Prism 6.0 h. Data in paired groups were analyzed using the paired student *t-*test. Sample differences among groups were analyzed using two-way ANOVA or unpaired student t-test. p < 0.05 was considered to have statistically significant difference.

## Additional Information

**Accession codes:** The RNA-seq original data reported in this paper have been deposited in the NIH GEO database (accession #. GSE85271).

**How to cite this article**: Cui, Z. *et al*. KANK1 inhibits cell growth by inducing apoptosis though regulating CXXC5 in human malignant peripheral nerve sheath tumors. *Sci. Rep.* **7**, 40325; doi: 10.1038/srep40325 (2017).

**Publisher's note:** Springer Nature remains neutral with regard to jurisdictional claims in published maps and institutional affiliations.

## Supplementary Material

Supplementary Information

Supplementary File 1
