# Corrigendum: KANK1 inhibits cell growth by inducing apoptosis through regulating CXXC5 in human malignant peripheral nerve sheath tumors

**DOI:** 10.1038/srep46392

**Published:** 2017-04-24

**Authors:** Zhibin Cui, Yingjia Shen, Kenny H. Chen, Suresh K. Mittal, Jer-Yen Yang, GuangJun Zhang

Scientific Reports 7: Article number: 4032510.1038/srep40325; published online 01 09 2017; updated on 04 24 2017

The original version of this Article contained an error in the title where:

“KANK1 inhibits cell growth by inducing apoptosis though regulating CXXC5 in human malignant peripheral nerve sheath tumors”.

Now reads:

“KANK1 inhibits cell growth by inducing apoptosis through regulating CXXC5 in human malignant peripheral nerve sheath tumors”.

There were also typographical errors in the legends of Figure 3 and Figure 5, where the Figure 3 legend:

“Quadrant I-IV represents dead cell population, early apoptotic cell population, late apoptotic cell population, and live cell population, respectively”.

Now reads:

“Quadrant I-IV represents dead cell population, late apoptotic cell population, early apoptotic cell population, and live cell population, respectively”.

In addition, the legend of Figure 5.

“Later apoptotic cell percentage was reduced from 67.5% to ~31% in both CXXC5 shRNAs infected cells”.

Now reads:

“Early apoptotic cell percentage was reduced from 67.5% to ~31% in both CXXC5 shRNAs infected cells”.

These errors have now been corrected in the HTML and PDF versions of this Article, and the Supplementary Information file that accompanies the Article.

