# Supplementary material for: KANK1 inhibits cell growth by inducing apoptosis though regulating CXXC5 in human malignant peripheral nerve sheath tumors
Source: Sci Rep. 2017 Jan 9;7:40325. doi: 10.1038/srep40325 (PMC5220314; doi:10.1038/srep40325)
Supplement: Supplementary Information [file srep40325-s1.pdf]

KANK1 inhibits cell growth by inducing apoptosis though regulating CXXC5 in human malignant peripheral nerve sheath tumors

Zhibin Cui[1], Yingjia Shen[2], Kenny H. Chen[1], Suresh K. Mittal[1,3,4], Jer-Yen Yang[3,5], GuangJun Zhang[1,3,4,6#]

SUPPLEMENTARY INFORMATION

Supplementary Figures

Figure S1. KANK1 frequent loss of DNA copy numbers in human and zebrafish MPNSTs.

(**a**). Heat map of human chromosome 9. KANK1 is located on chromosome 9p, which is found to be under-represented in 52 out of 61 human MPNSTs. Green line represents the *KANK1* gene locus, and the vertical black line indicates the centrosome of chromosome 9. (**b**). Both *kank1a* and *kank1b* are located at zebrafish chromosome 5, which is underrepresented in 60% of zebrafish MPNSTs. Samples are sorted top-to-bottom by decreasing deletion amplitude at the respective *KANK1*/*kank1* locus indicated by a green line. Blue and red bars at the right side of each panel indicate which samples with *KANK1*/*kank1* losses (blue) or gains (red). Color densities are corresponding to the degree of loss and gain as previously described[1]. (**c**). KANK1 gene mutations in human cancer genomes. *KANK1* gene analysis using cBioportal (http://www.cbioportal.org/). In the MSKCC 2014 data set (black arrow), 3 out of 15 MPNST samples (20%) had deep deletions and 5 out 15 samples (33%) had shallow deletions.

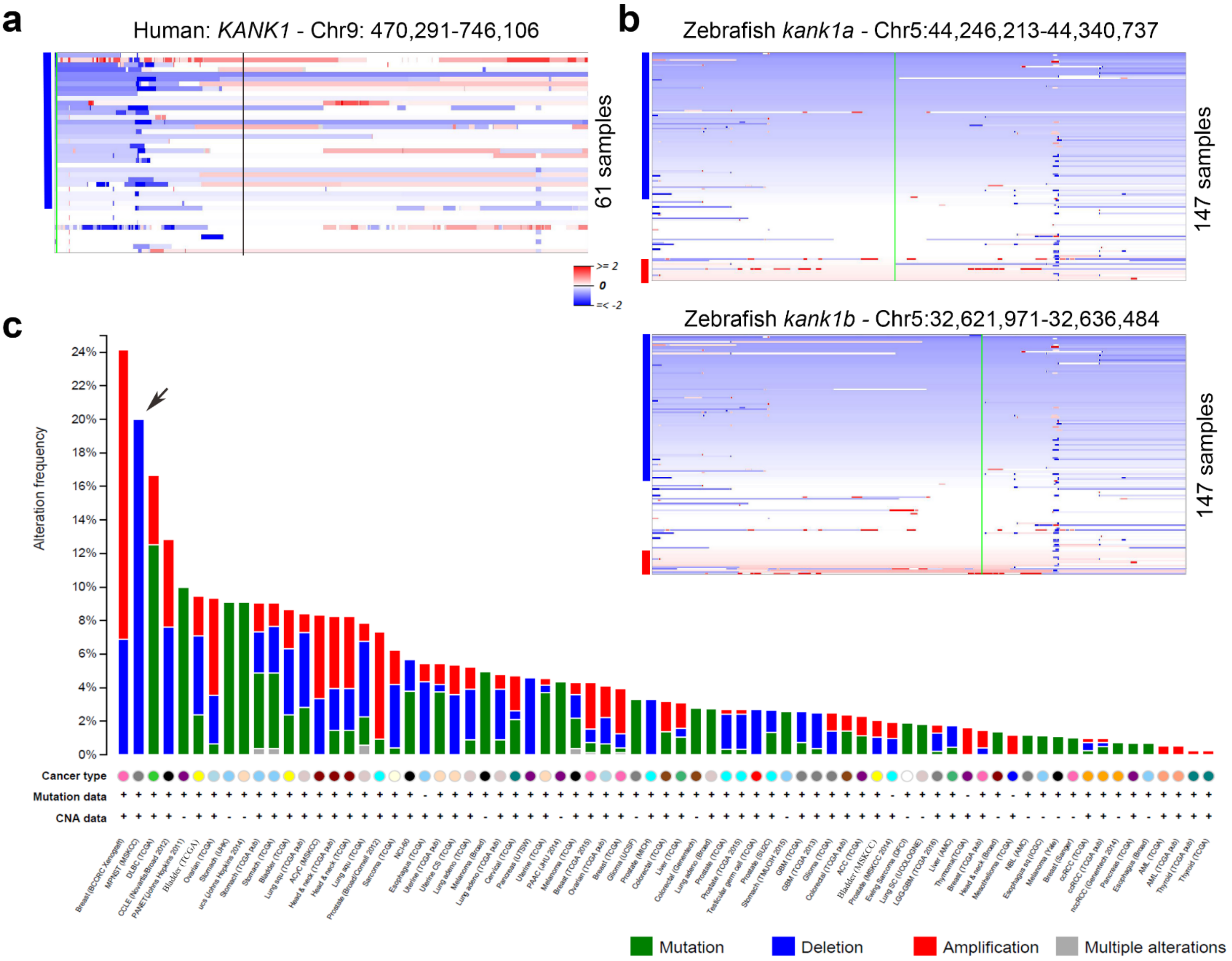

Figure S2. *KANK1* gene expression is tightly regulated by the dosage of doxycycline treatments. (**a**). KANK1 protein expression in STS26T (pLIX405-*KANK1*) cell is induced by doxycycline (+Dox) in a dose-dependent manner. ACTB, beta-actin control. (**b**). EGFP expression (fused at the C-terminus of *KANK1*) level (brightness) is also roughly corresponding to the dosage of the doxycycline under a fluorescence microscope. (**c**). KANK1 expression is reversible upon remove of doxycycline. Stable STS26T cells were treated with doxycycline at the first day and the doxycycline was withdrawn from culture medium at the fourth day. (**d**). MTT assay showed STS26T cell growth inhibition at different concentrations of doxycycline. (**e**). Plate colony formation assay with or without doxycycline at 1μg/ml. Cells were cultured in the medium with 0.5% FBS. Asterisk (*) shows statistical significance, p= 0.0063.

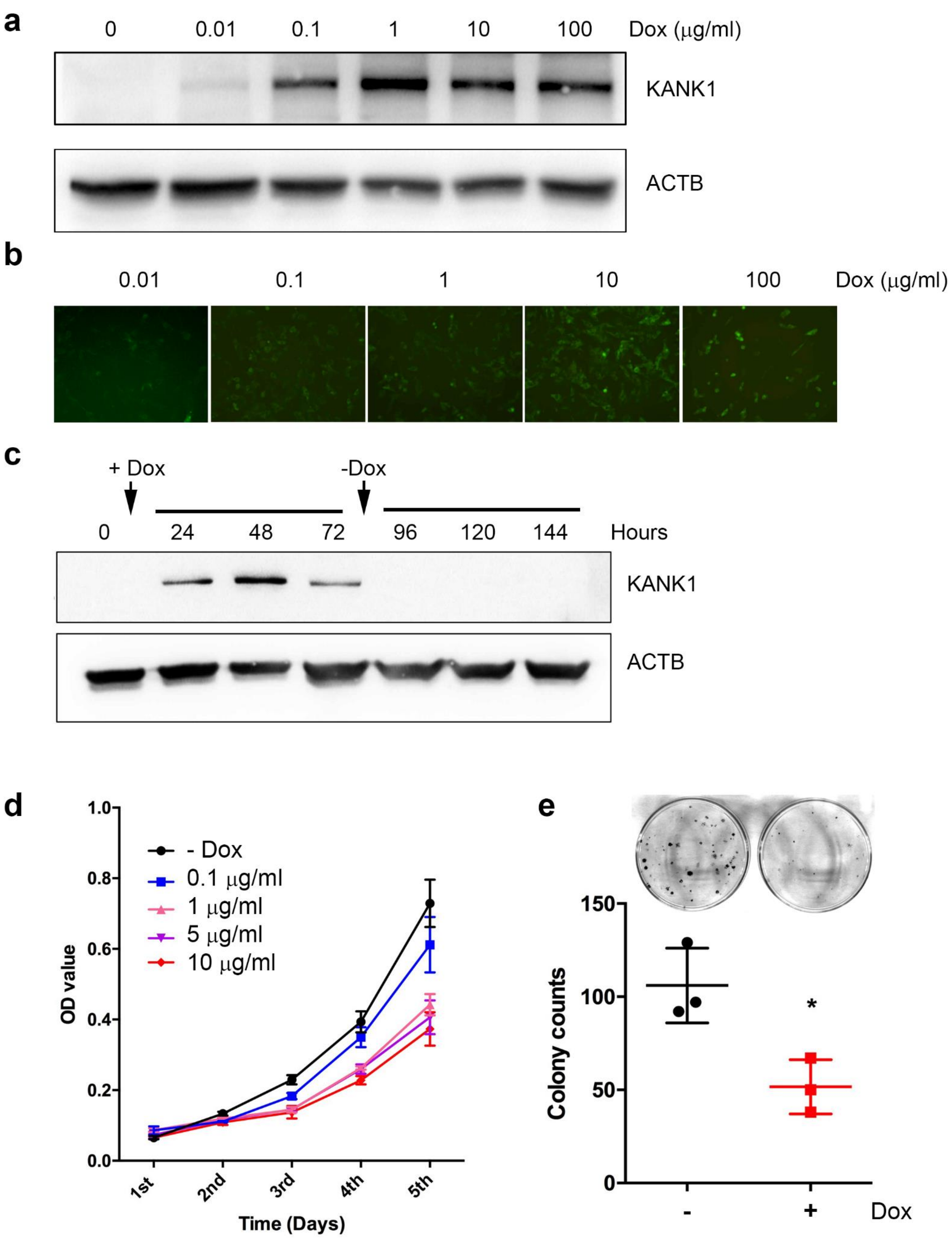
a
0
0.01
0.1
1
10
100
Dox (μg/ml)
KANK1
ACTB
b
0.01
0.1
1
10
100
Dox (μg/ml)
c
+ Dox
-Dox
0
24
48
72
96
120
144
Hours
KANK1
ACTB
d
- Dox
0.1 μg/ml
1 μg/ml
5 μg/ml
10 μg/ml
OD value
1.0
0.8
0.6
0.4
0.2
0.0
1st
2nd
3rd
4th
5th
Time (Days)
e
Colony counts
150
100
50
0
*
-
+
Dox

Figure S3. Doxycycline has no effect on cell growth of non-transfected MPNST cells. MTT assays were performed to evaluate the effects of doxycycline on non-transfected STS26T (**a**) and S462 (**b**) cells. Two dosages (1μg/mL and 5μg/mL) of doxycycline were examined. Student t-test was performed at each day. No statistical differences were detected as p is greater than 0.05 for any of the two conditions.

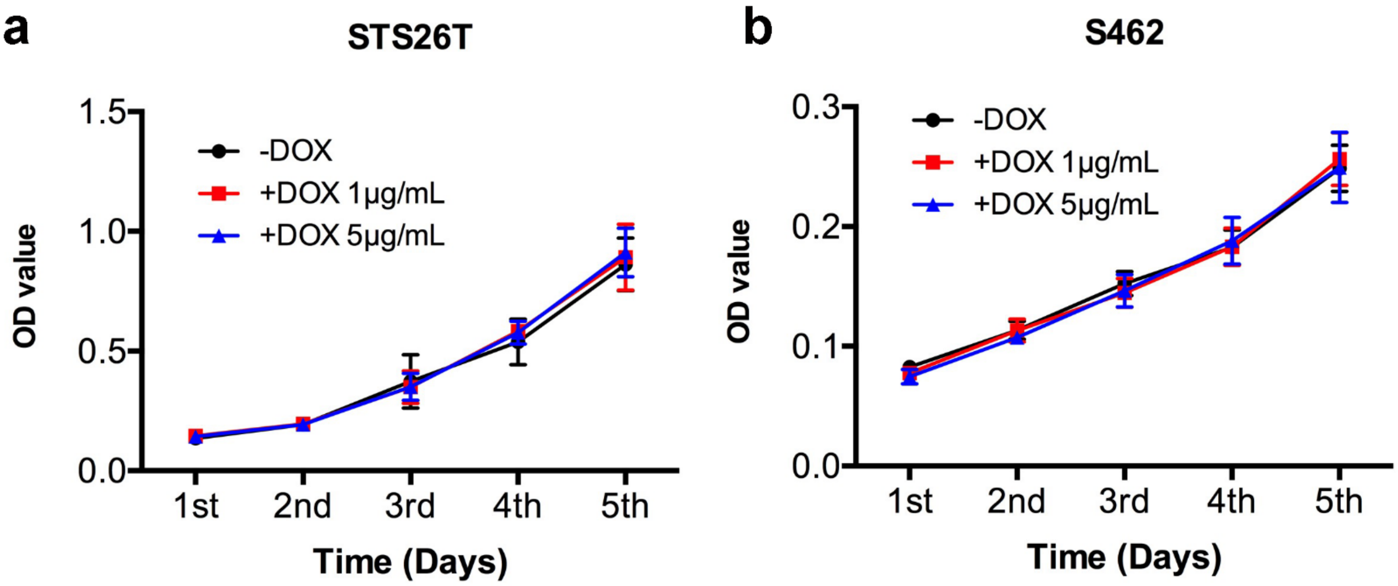

Figure S4. *KANK1* restoration did not lead to cell proliferation inhibition in human MPNST cells. Cells treated with or without doxycycline were measured at 48, 72, and 96 hours. Cell cycle properties were analyzed by flow cytometry analysis using BrdU for S phase labeling. KANK1 expressing STS26T cells and the control group were stained with anti-BrdU antibody (Y axis) and propidium iodide (X axis). No significant differences were observed in both 10% and 0.5% FBS culture conditions.

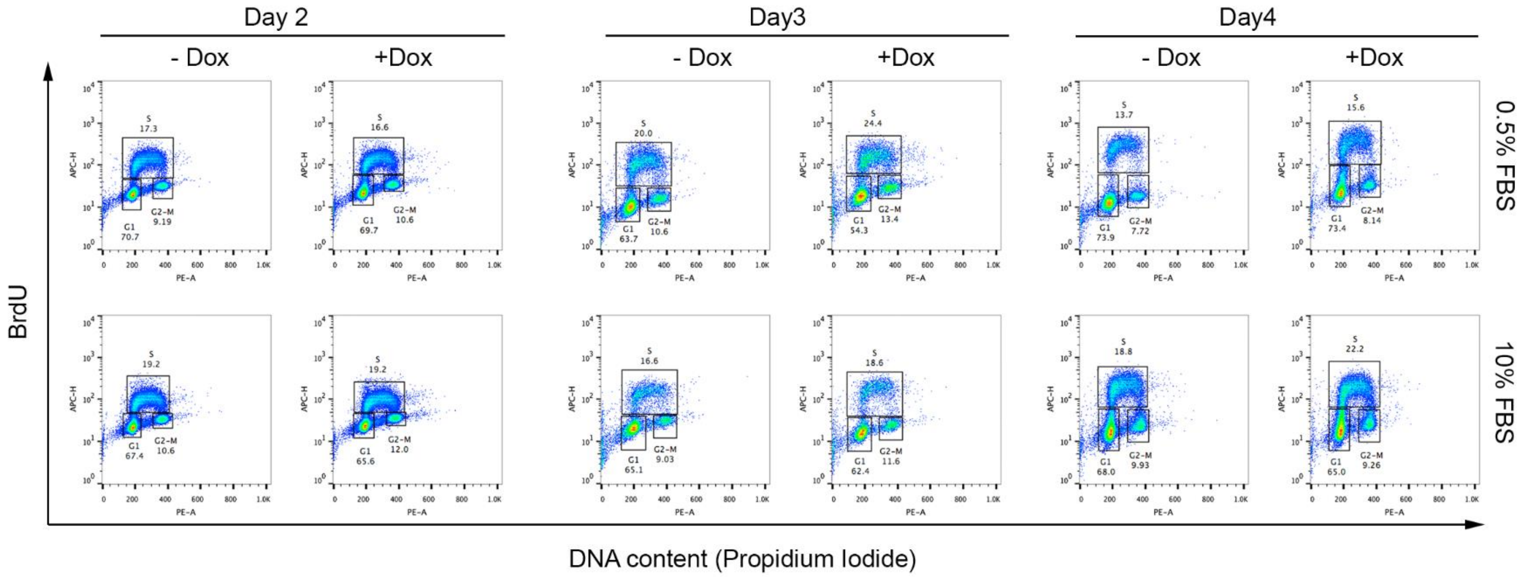

Figure S5. KANK1 restoration led to more apoptotic cells in xenografted tumors. Immunohistochemistry was performed using a cleaved caspase 3 antibody on both control (**a**-**f**) and KANK1 restored (**g**-**l**) tumors. Images were collected at X40 magnification. Scale bar is labeled in the panel **l**. All the slides were counter stained with hematoxylin. Positively stained cells (brown color) from these six pairs of microscopic fields were counted and analyzed in panel **m**. Asterisk (*) shows statistical significance, p= 0.005

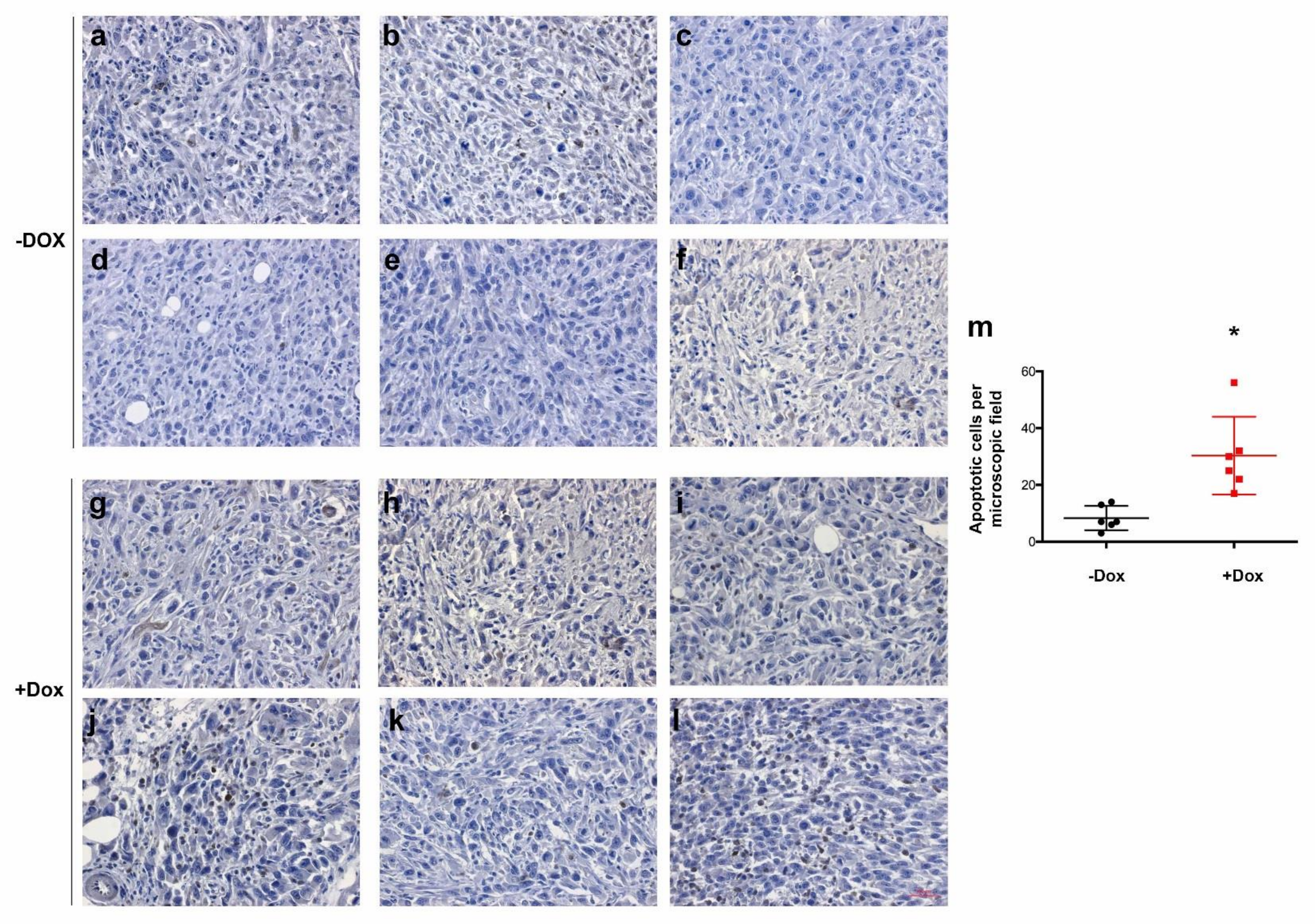

Figure S6. Cell nucleus morphology in KANK1-induced cells. Hoechst 33342 staining was performed to evaluate apoptosis in the KANK1-induced S462 stable cells without (**a**-**c**) and with doxycycline treatment (**d**-**f**) after 48 hours culture. Cells with condensed nucleus were counted as apoptotic cells. Typical apoptotic cells were labeled with white arrow heads in panels (**a** & **d)**, apoptotic rates were based on X cells counting. (**g**). The student t-test revealed the difference was significant (p=0.0052).

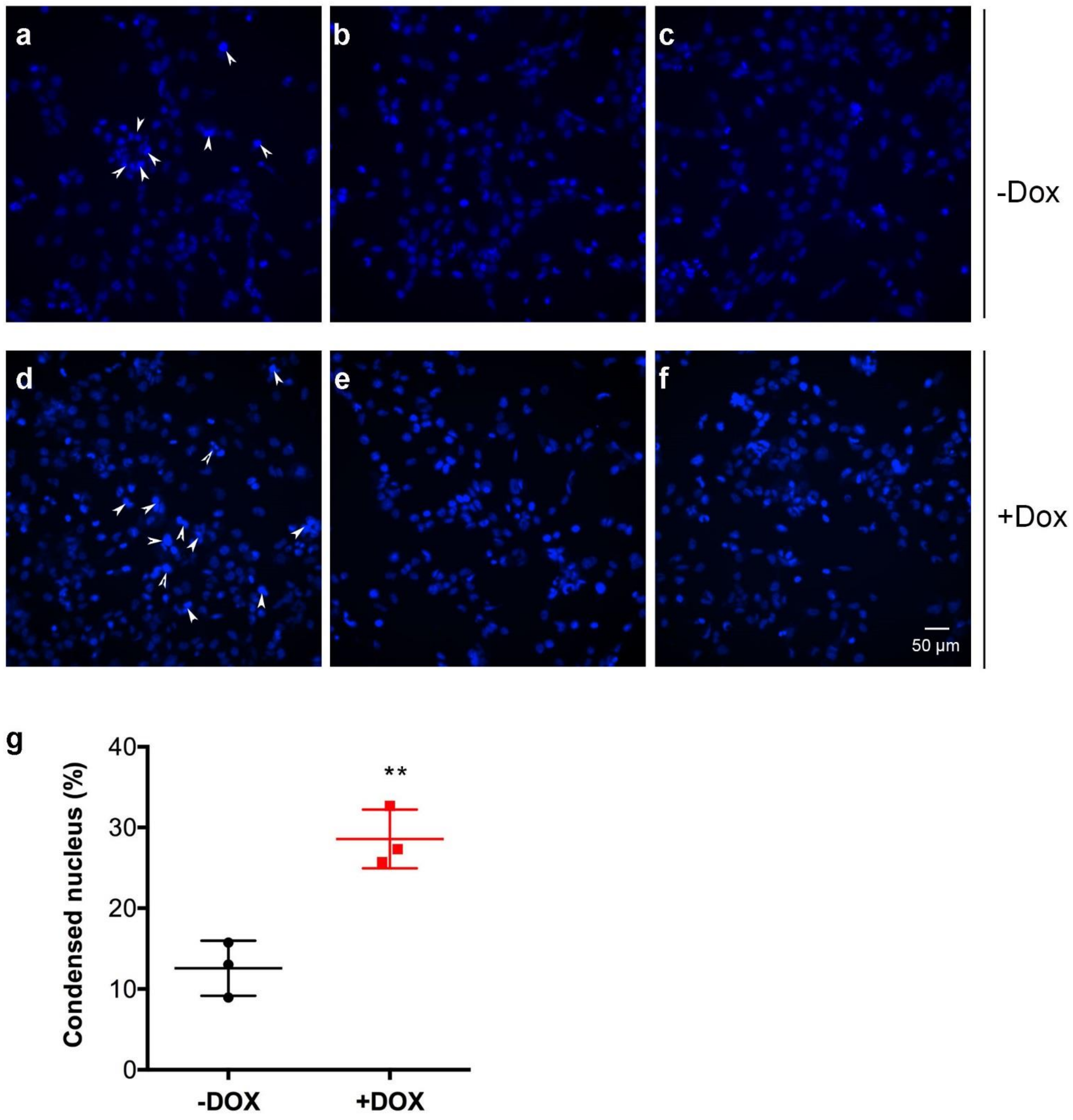

Figure S7. Transient overexpression of KANK1 upregulates CXXC5 in HEK293T cells. Transient transfections were performed using intact KANK1 and C-terminal GFP fusion KANK1 in HEK293T cells, respectively. CXXC5 protein levels were upregulated in both transfections. ACTB, beta-actin control.

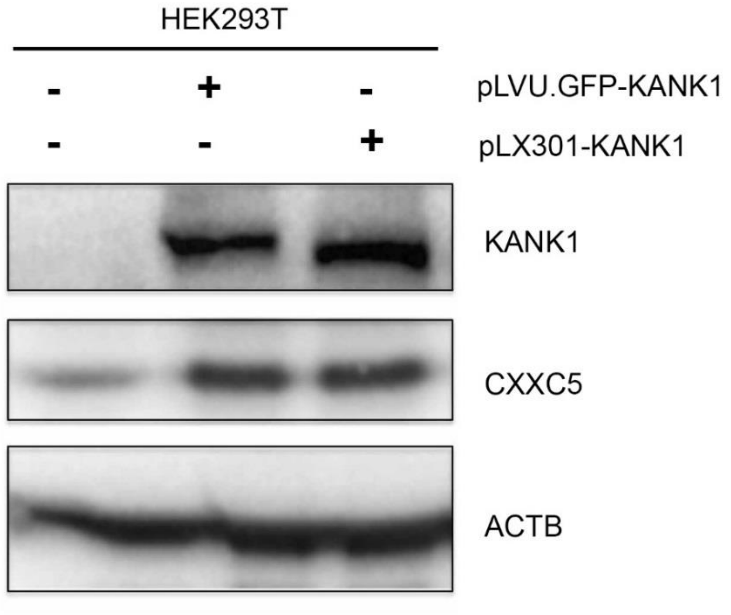

Supplementary Table

Supplementary Table 1. Primers used for quantitative RT-PCR

Human Genes	Forward Primer 5'->3'	Reverse Primer 5'->3'
ACTB	CTGGAACGGTGAAGGTGACA	AAGGGACTTCCTGTAACAATGCA
BAD	GGTAGGAGCTGTGGCGACT	CAAGCATCATCGCCAGG
CXXC5	CTTGGACGACTCGGAGAGC	AGGGTCCACAGACCAGGAC
FASTK	GGAGGTCTGAGCAGAGAGCA	GACTGAGGGAGCGACCTG
KANK1	CCAGATGTCCTCCGCTATGT	GCATGTCCTTCTCTGCTTCC
NME3	CTGCCGGGCTACTCATACAG	GATTTCTGCATCGAGGTTGG
SIVA1	CGTGAGCCAGAGGGAGTTGAG	GACTCTGGCAGGTGAACGACG

Supplementary File 1. Differentially expressed genes in KANK1-induced and –uninduced cells by RNA-seq analysis.

References

1. Zhang G, Hoersch S, Amsterdam A, et al. Comparative oncogenomic analysis of copy number alterations in human and zebrafish tumors enables cancer driver discovery. *PLoS genetics.* 2013; 9(8):e1003734.
